# Supplementary material for: Native American resilience to protect family nutrition during a pandemic: A qualitative analysis
Source: PLOS Glob Public Health. 2026 Mar 5;6(3):e0004822. doi: 10.1371/journal.pgph.0004822 (PMC12962456; doi:10.1371/journal.pgph.0004822)
Supplement: S1 Text — Full text of the script used to conduct the In Depth Interviews (IDI). (DOCX) [file pgph.0004822.s001.docx]

**IN-DEPTH INTERVIEW (IDI) GUIDE FOR PECO 2 PARTICIPANTS**

**COVID-19 IMPACT**

IDI IDENTIFICATION

| Location & Date of IDI | Site: ☐ Fort Defiance  ☐ Shiprock  ☐ Whiteriver  Location: __________________  Date: ____/_____/____ (MM/DD/YYYY) |
| --- | --- |
| Results of the IDI | Check one:  ☐ Complete  ☐ Incomplete, did not want to continue  ☐ Incomplete, respondent called away  ☐ Refused  ☐ No show  ☐ Other (specify): ___________________ |
| Time IDI began: ____: ____ (HH:MM) | Time IDI ended: ____: ____ (HH:MM) |
| Facilitator’s name: | Note-taker’s name: |

**PARTICIPANT ID NUMBER: FSN** ___ ___ ___ ___

CONSENT STATUS OF IDI PARTICIPANT: Consent date: ____/_____/____ (MM/DD/YYYY)

**Did this participant complete the COVID-19 Impact Questionnaire within their 18-month or 24-month assessment?**

☐ No. Complete the COVID-19 Impact Questionnaire before the IDI.

☐ Yes. Proceed to the IDI guide.

**INTRODUCTION/ PURPOSE**

- **Welcome –** Interviewer should introduce self (and note-taker if there is one)
- **Introduction/Overview** – “Today we will be discussing your experiences with food and drinking water access during the COVID-19 pandemic. You were selected because you have participated in the Family Spirit Nurture Program. We are interested in knowing your thoughts on what may be helping or preventing access to food and safe drinking water during the pandemic. We are also interested in your thoughts about sugary drink consumption during the pandemic, and how your participation in the study was impacted by the pandemic. Your responses to our questions will help us better understand how the pandemic might have changed food and drinking water access on Navajo and how to better help families with these changes in the future. There are no right or wrong answers. We would like your honest opinions.”
- **Permission to Audio Record** – “In order to ensure we do not miss any of your comments, we would like to record this interview. We will be unable to write fast enough to record all the information you share with us. We will not use your name in our reports. Are you okay with us recording the interview today?”
- **Answer Questions** - “Do you have any questions for me?”
- **Permission to Begin -** “May I turn on the recorder now to begin the interview? If at any time you would like to say something that you would not like me to record, please just tell me and I will turn off the audio-recorder.”

**INTERVIEW QUESTIONS**

This interview is broken up into three sections. The first section is about food and drink access and will begin with questions about you and your child’s experiences before the pandemic – since March 2020. Then we will ask similar questions about you and your child’s experiences during the start of the pandemic (March 2020). To help our conversation, I calculated how old your child was in March 2020 and I will remind us throughout the interview what time period we are talking about. According to my calculations, your child is now __________. Is that right?

That means your child was about ______ at the start of the pandemic (March 2020). Does that also sound right?

**Great, let’s first talk a little about your child who was in the study with you.**

- 1. **Child’s food and drink access BEFORE the COVID-19 pandemic**

**Let’s start with talking about before the pandemic. When your child was __________. We will only be asking about the time when your child was _________ until ____________.**

[Ask some general warm-up questions to help frame the discussion for the participant, such as: What was your child eating then? What things were they drinking at that age?]

- 1. Before the pandemic, so before March 2020, were you a part of any food programs (WIC, SNAP, etc.)?

**Probe:** Did you receive any commodity food, food boxes, or go to a food pantry?

**Now I’d like to hear what you think and know about how you accessed food for your child before the COVID-19 pandemic.**

- 1. Where was the main place you got your child’s food before the COVID-19 pandemic?

**Probe**: particular stores, towns, relatives, churches, etc.

How far away is this food source?

What was your transportation like?

What did you think about the price of this food?

How often do you have to go get food?

Before the pandemic, was it ever challenging to get the food you wanted for your family?

- 1. How did you feel about the food your child ate before the COVID-19 pandemic?

**Probe**: amount, types of food (healthy/less healthy)

*[Only ask 1.4-1.6 if the child was born before March 2020]*

- 1. What was your main source of **water for your child to drink or for formula** before the COVID-19 pandemic, so before March 2020?

**Probe**: particular stores, towns, relatives, churches, etc.

What was your transportation like?

What did you think about the price of this water before the pandemic?

How often would you have to go get water?

Before the pandemic, was it ever challenging to get drinking water for your family?

- 1. Before the COVID-19 pandemic, so before March 2020, what drinks did your child drink other than water, milk, and formula?

**Probe:**  What do you think about the amount of ________ your child was drinking? What do you think is a healthy amount of _______?

- 1. How did you feel about the drinks your child had before the COVID-19 pandemic?

**Probe**: amount, types of drinks (healthy/less healthy)

- 1. **Child’s food and drink access during the COVID-19 pandemic**

**Now, we’re going to switch to questions about access to food programs DURING the pandemic (since March 2020). Just a reminder: your child was _______________ at the start of the pandemic.**

- 1. Since the pandemic started (March 2020), Have you been a part of any food programs (WIC, SNAP, etc.)? **PROBE**: Have you received any food commodities food boxes, or gone to a food pantry?
  2. Are you still receiving services from these programs now?
  3. Did your experience with any of these programs change during the COVID-19 pandemic?

**Probe**: access to the programs, more or less services during the pandemic, benefit changes

**Now I’d like to hear what you think and know about how the COVID-19 pandemic affected the food you could get for your child who was in the study with you.**

- 1. What have been the main places you get your child’s food during the COVID-19 pandemic and lockdowns, since March 2020?

**Probe**: particular stores, towns, relatives, churches, etc.

*[If participant’s food sources were different than before the pandemic – answer to 1.2]*

How far away was this food source?

What was your transportation like?

What did you think about the price of this food?

How often did you have to go get food?

Were there any challenges getting food this way?

*[If participant’s food sources are the same before and during the pandemic]*

Did the hours or days you could get this food change because of COVID-19 or the lockdown?

Do you think differently about the prices at this food source/location during the pandemic?

Did how often you go get food change during the pandemic?

Are there any new challenges getting food this way?

- 1. How do you feel about the food your child has been eating during the COVID-19 pandemic?

**Probe**: amount, types of foods (healthier/less healthy foods)

- 1. What foods do you think are important for your child to eat?

Probe: Ideally, what foods do you want to feed your child?

- 1. Do you think COVID-19 has changed your ability to feed your child what you want to?

**Probe**: types of foods (healthier/less healthy foods), availability of foods

**Was your child eating solid foods before the pandemic, before March 2020? [Reminder that your child was ___________ in March 2020]**

*[Ask 2.7-2.10 only if the child was eating solid foods before the pandemic]*

- 1. In what ways has COVID-19 changed your child’s eating habits, if at all?

**Probes**: Did the COVID-19 pandemic change how or when your child eats?

- different mealtimes, more time to feed the child, less time to feed the child
- Did the COVID-19 pandemic change what your child eats?
- changes in food sources, types of food not available, more time to feed the child, less time to feed the child
  1. Were some foods for your child easier to get during the COVID-19 pandemic?

Probe: Did this change over the course of the pandemic?

What are some examples?

- 1. Were some foods more difficult to get during the COVID-19 pandemic?

**Probe**: Did this change over the course of the pandemic?

What are some examples?

*[Ask everyone the questions below]*

**Now I’d like to hear what you think and know about how the COVID-19 pandemic affected the drinks you could get for your child who was in the study with you.**

- 1. What has been your main source of **water for your child to drink or for formula** during the COVID-19 pandemic?

**Probe**: particular stores, towns, relatives, churches, etc.

What do you think about the price of this water?

[*If they leave the house for water]*

How far away is this water source?

What is your transportation like?

How often do you have to go get water?

Are there any challenges getting water this way?

**Probe**: Closed stores/limited hours, closed churches, stay at home orders, social distancing and isolation

- 1. What drinks does your child drink other than water, milk, and formula?

**Probe:**  What do you think about the amount of ________ your child drinks? What do you think is a healthy amount of _______?

- 1. How do you feel about the drinks your child has been drinking during the COVID-19 pandemic?

**Probe**: amount, types of drinks (healthy/less healthy)

- 1. What drinks do you think are important for your child to drink?

Probe: Ideally, what do you want to give your child to drink?

- 1. Do you think COVID-19 has changed your ability to give your child the drinks you want to give them?

*[Only ask 2.15 – 2.16 if the child was born before March 2020]*

- 1. Did what your child drinks change during the COVID-19 pandemic?

[If participant answers ‘Yes’] Why did it change during COVID-19?

**Probe**: changes in drink sources, available drinks

- 1. Were some drinks for your child easier to get than others during the COVID-19 pandemic?

Probe: water, milk, soda, juice, etc., availability of different types of drinks

- 1. Were some drinks for your child more difficult to get than others during parts of the COVID-19 pandemic?

**Probe**: water, milk, soda, juice, etc. , availability of different types of drinks

- 1. **General questions about COVID-19 impact**

**Let’s now talk a little about how the COVID-19 pandemic affected you and your family in general.**

- 1. In general, thinking back on the pandemic, what helped you to get access to food and drinking water? Was there anything that prevented you from getting access to food and drinking water?
  2. In general, what do you wish that the community could have offered? Any services or things that could have been different to help you get access to food and water?
  3. Did the COVID-19 pandemic or any of the lockdown policies introduce new challenges for you or your family?

[If participant answers ‘Yes’] What were the new challenges?

What did you do to respond to these challenges?

- 1. Did the COVID-19 pandemic or any of the lockdown policies make any existing challenges for you and your family more difficult?

[If participant answers ‘Yes’] What were the challenges?

What did you do to respond to these challenges?

**4. Questions about participant’s experience in the study during COVID-19**

**Now I’d like to talk to you about what it was like being in this study and how the COVID-19 pandemic impacted your participation.**

*[For intervention group only]*

- 1. Were any of the lessons or key messages you learned from our staff (both before and during the pandemic) especially meaningful or memorable?

[If participant answers ‘Yes’] Which ones? Why were they memorable or meaningful? What are the three biggest take aways from the lessons you received?

- 1. Were there any lessons you would have preferred to skip?

[If participant answers ‘Yes’] Which ones? Why would you have preferred to skip them?

- 1. Are there things that Family Spirit Nurture teaches that you think are especially doable during times like the COVID-19 pandemic?
  2. Are there things that Family Spirit Nurture teaches that you think are especially hard to do during COVID-19 times?

**Share list of FSN lesson titles to jog memory and ask questions again.**

*[For participants who were in the study during COVID-19]*

- 1. What were some challenges with completing the lessons during COVID-19?

**Probe**: How could the study team make that easier?

- 1. What were some challenges with completing the surveys during COVID-19?

**Probe**: How could the study team make that easier?

- 1. What did you enjoy about completing the lessons during the COVID-19 pandemic?
  2. What did you enjoy about completing the surveys during the COVID-19 pandemic?
  3. How did you feel about completing lessons by phone or video during the pandemic? How does this compare to in-person lesson visits before the pandemic?
  4. How did you feel about completing surveys by phone or video during the pandemic? How does this compare to in-person survey visits before the pandemic?

4.11 What suggestions do you have for how we can improve this program for

other families in the future?

**WRAP-UP OF IDI**

- The interviewer should **very briefly** summarize key points from the discussion, and then ask:
  - Is there anything else you would like to add?
- THANK THE IDI PARTICIPANT. Distribute a gift card and have the participant initial the gift card log to confirm they received the gift card.
